# Empowering patients to choose and providers to improve. A focus group study on quality information needs in Switzerland's in- and outpatient somatic care

**DOI:** 10.1016/j.hpopen.2026.100191

**Published:** 2026-10-16

**Authors:** Carla Walker, Maxime Sapin, Sarah Mantwill, Justus Vogel, David Ehlig, Alexander Geissler

**Affiliations:** a Chair of Health Economics, Policy and Management, School of Medicine, University of St. Gallen, St.-Jakob-Strasse 21, St. Gallen, CH-9000, Switzerland; b Department of Health Sciences & Medicine, University of Lucerne, Switzerland

**Keywords:** Public reporting, Quality monitoring, Somatic care, Focus groups

## Abstract

**Background:**

Quality monitoring ensures quality standards and improvement, while public reporting aims to inform patient choice. In Switzerland, quality monitoring and public reporting have fallen short of stakeholder expectations due to a misalignment with needs.

**Objectives:**

This study explores stakeholder needs in inpatient and outpatient care for public reporting and quality monitoring in Switzerland.

**Methods:**

In 2023, nine focus groups with 50 stakeholders from in- and outpatient somatic care were conducted. The data underwent qualitative content analysis to identify themes related to the purpose, challenges, and drivers of public reporting and quality monitoring, as well as indicator selection criteria.

**Results:**

Public participants emphasised accessible quality information to guide decisions, especially in chronic or serious illness. A major gap was a single point of contact for navigating care and shared decision-making. Public and expert views diverged on who should access and interpret quality data. Experts prioritised monitoring for internal improvement. Both groups cited administrative burden and system fragmentation as key barriers.

**Conclusion:**

While patients seek more involvement and access to data, expert reluctance to share quality information persists. Advancing public reporting requires recognising the public as a core audience and addressing data challenges through a national strategy.

## Highlights

• Quality monitoring and public reporting fall short of stakeholder expectations.
• Nine focus groups with 50 Swiss stakeholders in in- and outpatient somatic care.
• Patients demand quality transparency, not least to support shared decision-making.
• Barriers, like burdensome data collection, outweigh existing facilitators.
• A national strategy is needed to engage the public and address data challenges.

**Research in context**

What is already known about the topic? While there is evidence that the utilisation and acceptance of public reporting and quality monitoring remain low, there is little evidence on what stakeholders actually need in terms of quality information.

What does this study add to the literature? This study adds value by providing a detailed analysis of the current quality information needs of stakeholders in inpatient and outpatient somatic care in Switzerland, focusing on public reporting and quality monitoring. We gathered insights from 50 stakeholders, including patients, relatives, health care providers, regulators, payers, and other experts. This diverse range of perspectives offers a more holistic view of the challenges and opportunities for improving the transparency and effectiveness of quality information in health systems.

What are the policy implications? The combined findings from this study and existing literature highlight that patients are often not seen as the main target group for public reporting. However, patients express a strong desire for more transparency and wish to be more actively involved in decision-making regarding their health. This aligns with a growing emphasis on patient-centered care. Providers, depending on whether they work in inpatient or outpatient care, have different experiences with quality measurement and, consequently, prioritise different quality indicators. These differences should be considered when designing national quality strategies and quality monitoring systems.

## 1 Background

Quality of care varies across countries, regions, and providers 1., 2., 3.. Measuring, reporting, and comparing quality are perhaps the most important steps toward addressing variation and rapidly improving quality [4]. Policymakers are therefore developing systematic ways to measure and report quality information, aiming to empower patients to make better-informed decisions and providers to learn and improve 5., 6..

Quality monitoring is the systematic, continuous measurement, analysis, and reporting of health quality, typically using quality indicators, to assess performance against standards and provide providers and regulators with the feedback they need for quality assurance and improvement 7., 8., 9., 10.. Regulators are often legally mandated to oversee quality of care and thereby implement quality monitoring using structural, process, and outcome quality indicators, with the aim of ensuring high-quality standards and improving underperforming areas 11., 12.. At the same time, providers have an inherent interest in monitoring their own quality, as providing high-quality services, facilitated by quality monitoring, can serve as a competitive advantage 11., 13..

However, within quality monitoring, quality indicators are often seen as inadequate, the documentation process burdensome, and stakeholder acceptance, particularly among providers, low 10., 11.. Additionally, the number of quality indicators has grown considerably in recent years, increasing the complexity of selecting those effective for monitoring provider quality and patient outcomes [14].

Another important instrument in this context is public reporting, which aims to ensure accountability by publishing quality information to enhance transparency and support patient choice of providers [9]. In theory, enabling patients to choose providers based on quality information could contribute to overall system improvement [15]. While patients express a need for such information to make informed choices 16., 17., utilisation of public reporting remains low 9., 18.. One issue appears to be that available quality information is often inaccessible or difficult to interpret. While earlier studies suggest that an overload of data can overwhelm patients [18], more recent research indicates that the lack of relevant, well-structured, and comprehensible information is a key barrier 6., 19.. This suggests that both, the type of information presented and how it is communicated, are critical factors in improving public engagement 1., 20., 21., 22., 23.. Moreover, the lack of accessible, comparable quality information limits consumers' ability to make informed choices, potentially constraining the effectiveness of pro-choice policies. We hypothesise that the low utilisation of public reporting is because the right information is not being presented in the right way and does not reach all user groups equally well 10., 24.. The differing objectives and information needs of stakeholders result in conflicting requirements for quality monitoring and public reporting [25]. Despite their importance, a systematic comparison of these needs across stakeholders has not been extensively explored.

Switzerland is an example of a country which fundamentally reformed its health system by combining competition-based instruments with supply-side regulation. Faced with rising health care costs, the Federal Assembly approved the revision of the Health Insurance Act (KVG) on 21 December 2007, marking one of the most important amendments to the KVG since its introduction in 1996. The goal was to curb cost growth in inpatient somatic care while maintaining access to high-quality treatment. To realise the goal, quality-based competition between hospitals should be enhanced through increased transparency and greater choice for insured persons. At the same time, cantonal hospital planning was formalised to better steer services [26].

A 2019 assessment of the KVG reform identified several areas for optimisation. On the demand side, the public has made limited use of the expanded hospital choice. Patients often do not understand what quality entails or lack access to transparent, understandable quality data. This lack of understanding results in the underutilisation of the extended hospital choice available to insured individuals. The report also highlights that the cantons make little use of data in hospital planning to stimulate competition. This stems from the cantons' broad interpretation of the criteria for quality, as well as a partially inadequate data basis [26]. The 2019 revision of the Federal Health Insurance Act (KVG) then aimed to strengthen quality and cost-effectiveness and established the Federal Quality Commission (FQC) [27]. In its quality development goals for 2022–2024, the Federal Council mandated the FQC to design a national quality monitoring and public reporting system. To clarify who should use these instruments and for what purposes, the FQC commissioned the Swiss Learning Health System, a consortium of several universities, to develop the conceptual foundations through a stakeholder dialogue between December 2022 and August 2023. The present study was conducted as part of this project and aims to address this gap by examining stakeholders' quality information needs in inpatient and outpatient somatic care in Switzerland. Specifically, we seek to answer the following research questions:

I. What quality information needs exist for different stakeholders in in- and outpatient somatic care in Switzerland?
II. What do stakeholders perceive as facilitators and barriers to quality monitoring and public reporting?
III. What factors need to be considered from the perspective of the stakeholders when selecting quality indicators for quality monitoring and public reporting in Switzerland?

By addressing these questions, we aim to provide a foundation for improving quality monitoring and public reporting in Switzerland and beyond. Understanding stakeholder needs and barriers will contribute to refining these frameworks and enhancing their effectiveness. In this study, we differentiate between public reporting, which disseminates quality information to the public, and quality monitoring, which involves sharing information between providers, regulators, and other stakeholders for oversight, benchmarking, and improvement [11].

## 2 Methods

The study is reported in accordance with the Consolidated Criteria for Reporting Qualitative Research (COREQ) 32-item checklist to ensure transparency and reliability (supplemental material 1) [28].

### 2.1 Focus groups

We conducted nine focus groups between May and July 2023, with five groups focusing on inpatient and outpatient somatic care. These included three groups with experts in inpatient care, two in outpatient care, and four with medical laypersons (for a detailed composition of the focus groups, see Table 1). The groups took place in conference rooms in Berne, Lausanne, Zurich, and St.Gallen, in both Swiss German (6/9) and French (3/9), ensuring broad geographic and cultural representation.

**Table 1:** Overview of focus groups and participants.

	Outpatient somatic care	Inpatient somatic care	Public
Number of focus groups	2	3	4
Total participants	10	16	24
Number of providers	4	5	0
Number of regulators	0	4	0
Number of payers	1	1	0
Number of association representatives	4	5	0
Number of academics	1	1	0
Number of individuals classified as patients with a chronic illness	0	0	16
Number of individuals classified as patients who have undergone surgery or given birth in the last 6 months	0	0	12
Number of individuals classified as relatives	0	0	13

Separate groups for the public aimed to reduce potential reluctance in sharing opinions alongside health care experts. For the expert focus groups, purposive sampling was employed to strategically select organisations and individuals with specific knowledge, expertise, or experience relevant to quality monitoring and public reporting. For the public focus groups, a combination of personal networks and targeted Facebook advertisements was used to recruit participants. Unlike the expert groups, where purposive sampling was applied, recruitment for the public focus groups aimed to ensure a diverse range of perspectives.

The expert focus groups involved 26 participants, including nine from the provider group (public and private in- and outpatient care), four regulators, two payers, nine from Swiss health associations, and two academics (see supplemental material 2 for more information on the expert focus group).

The public focus groups had 24 participants, including individuals with chronic or acute illnesses, those who had recently undergone surgery or given birth, and their relatives (for a detailed composition, see Table 2). Participants could have multiple roles. One participant with a chronic illness discontinued participation midway through the focus group due to feeling unwell.

**Table 2:** Characteristics of the public focus group participants.

Total number of participants of the public focus group		24
Gender	Female	20
	Male	3
	Neutral	1
Age groups	18–30	1
	31–40	3
	41–50	8
	51–60	7
	61–70	5
Residence (Canton)	Vaud	11
	St.Gallen	9
	Zurich	2
	Berne	2
Health-related (multiple responses possible)	Chronic disease	16
	Acute illness and/or surgery in the last 6 months	12
	Active in a patient organization	3
	Relative of a patient (suffering from chronic illness, acute illness, or elective surgery)	13

Focus groups lasted 1 h 40 min for experts and 1 h for the public, with one moderator and one notetaker (see supplemental material 3 for more information on the research team and the reflexivity). Facilitators moderated the session and noted down important information on flipcharts. The focus group questions were shaped by the aims of the project. The draft questions were reviewed by the authors to test their appropriateness for the intended audiences and modified based on their input. For the public, two leading questions were posed during the focus groups: *(1) What does ‘good quality’ in health care mean to you?* and *(2) Would you like quality information to be published? If yes, what kind of quality information should be published?* For experts, the leading questions were: *(1) From your perspective, what quality information needs to be monitored and reported in Switzerland? (2) What are the most important criteria for selecting quality indicators?* To explore these topics further and facilitate the discussions, moderators used additional prompts (see supplemental material 4).

### 2.2 Data processing and analysis

Focus group discussions were recorded, transcribed using Sonix.ai (version 2024), and manually cleaned. Data was analysed in the original language to preserve cultural nuances. The codes and the codebook were defined in English, however, allowing the research team to view the data set as a whole. The data underwent qualitative inductive content analysis, where the content was systematically interpreted through coding, memo-writing and the identification of themes and patterns [29]. One author inductively generated codes and code groups from initial reading of the text, which were then defined and used to develop a codebook that the research team reviewed and finalised. By independently double-coding two French and two German transcripts, we checked intercoder reliability. The authors continued coding the other transcripts line by line in their respective native languages using the application *Atlas.ti (Version 24.1)*. The coders worked together to arrange the findings into categories, from which they subsequently developed broader themes. Illustrative quotes supported the observations and strengthened confirmability. Throughout the coding process, the team continually compared categories and themes to maintain analytical rigour [30]. Data saturation was discussed among the research team during analysis. Thematic consistency was observed within the public, inpatient and outpatient care focus groups, indicating that key concerns were adequately explored within each stakeholder perspective. Results are presented using spider diagrams to illustrate theme importance. Participant quotes are labelled by focus group and participant type.

The study describes participant characteristics and the study context to support the transferability of findings to other settings. The documentation of study methods, interview questions, and the analysis approach supports dependability.

### 2.3 Ethical considerations

The project does not fall within the scope of the Swiss Federal Act on Research involving Human Beings (Human Research Act, HFG). Therefore, formal ethical approval was not required for this study. All methods were conducted in accordance with relevant institutional and national guidelines and regulations and with the ethical principles outlined in the Declaration of Helsinki.

Participants received detailed information about the study's purpose, procedures, and data use, including the recording of discussions and measures to ensure confidentiality. Participation was voluntary, and participants were informed of their right to withdraw at any time without consequences. Verbal informed consent was obtained from all participants prior to participation.

### 2.4 Patient and public involvement

Patients, relatives and the public were involved in this study as participants in four of the nine focus groups. They contributed to identifying key priorities and challenges in public reporting from a lay perspective. While members of the public were not involved in the initial development of the research questions or study design, the findings were shared with participants through a summary report, which is now publicly available [31]. This approach also demonstrates credibility, as participants were invited to provide feedback on the accuracy of preliminary insights at the conclusion of the focus group [32].

## 3 Results

The focus groups discussed various topics related to quality indicators, barriers, facilitators, and the purposes of quality monitoring and public reporting (see Fig. 1). Fig. 1 shows the different themes in a spider diagram, how often these codes were used and the profiles of the public and the in- and outpatient somatic care stakeholders.

**Fig. 1:**
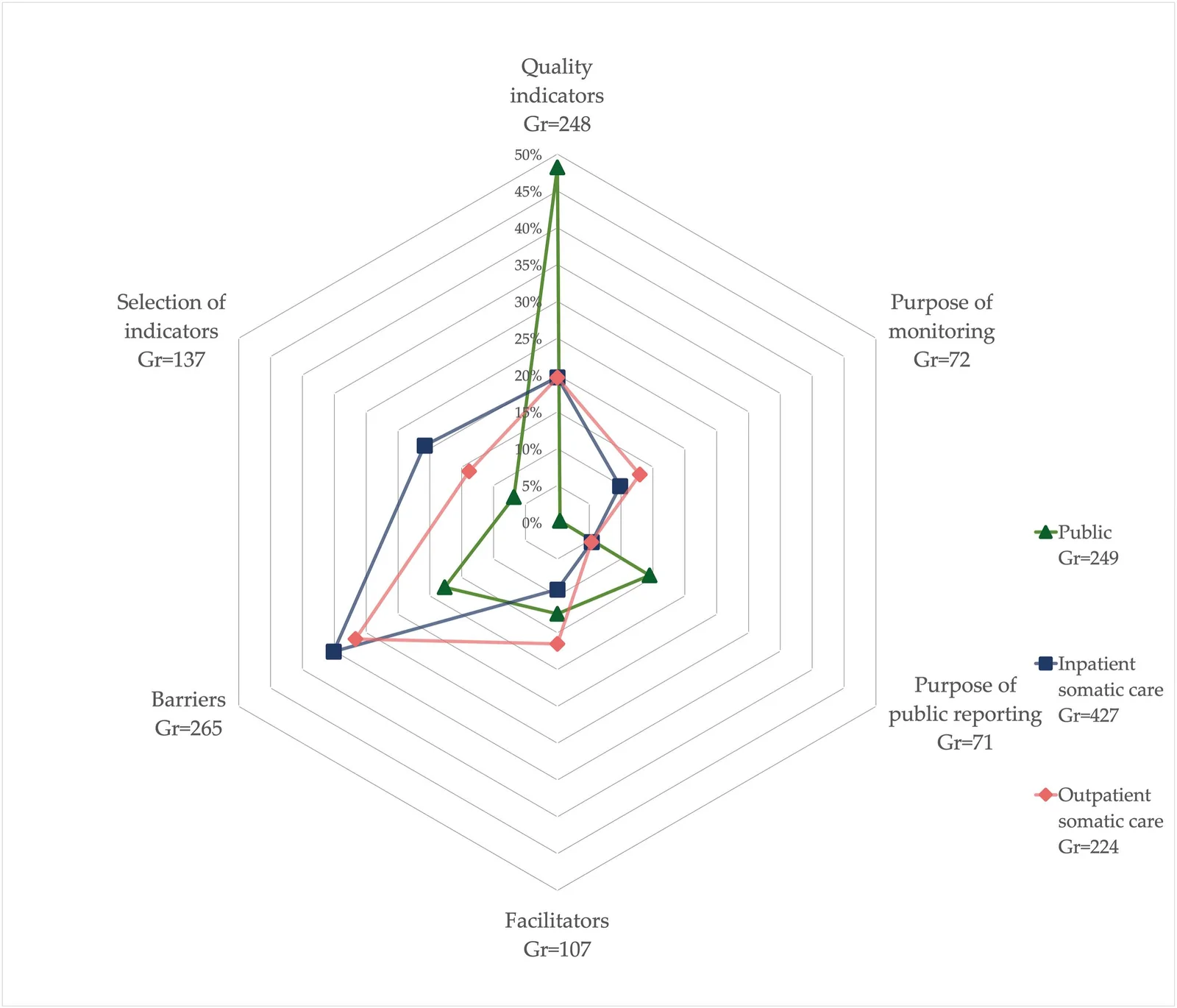
Overview of code groups displayed as a percentage of all topics discussed by focus groups. Note: Percentages represent the frequency of this theme relative to all topics discussed. Gr (Groundedness): Number of citations encoded by a code.

In assessing *quality indicators*, we examined the information needs of different stakeholders, with a particular focus on indicators for availability, structure, process, outcome, and patient-reported experience quality [7]. The two themes, *purpose of quality monitoring* and *purpose of public reporting,* capture statements regarding the rationale for quality monitoring or public reporting, respectively. *Facilitators* describe factors that enable or support the implementation of quality monitoring and public reporting, whereas *barriers* represent challenges or obstacles that hinder them. Lastly, *selection of quality indicators* refers to the criteria that participants thought were important to consider when choosing indicators.

As a percentage of all topics discussed, public participants emphasised quality indicators, barriers to public reporting, and shared decision-making (a detailed composition can be found in supplemental material 5). In contrast, inpatient and outpatient groups focused on barriers and selecting quality indicators. Both expert groups had minimal discussion on the purpose of public reporting.

### 3.1 Quality information needs

#### 3.1.1 Public focus groups

The public is particularly interested in availability and structural quality information (see Fig. 2), such as provider capacity, proximity, staffing, and qualifications. They also valued shared decision-making and Patient Reported Experience Measures (PREMs) and Outcome Measures (PROMs), though not all were familiar with these terms. A more detailed composition can be found in supplemental material 5 (Figure A 1).

**Fig. 2:**
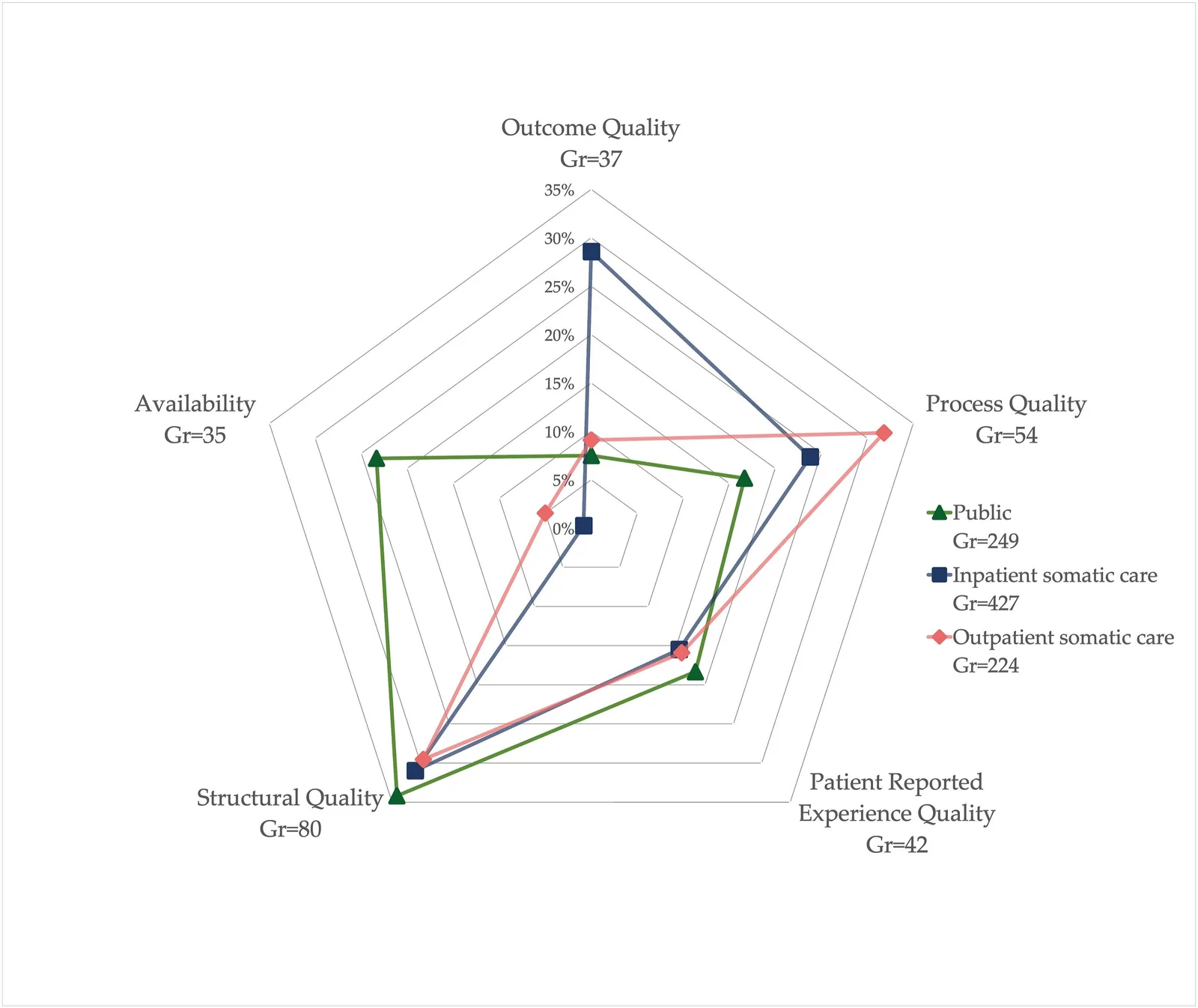
Overview of quality indicator groups displayed as a percentage of the quality indicator theme, discussed by focus groups. Expert focus groups*.*

Experts expressed interest in quality information across all five quality dimensions. PROMs and PREMs were seen as vital in patient-centred care, particularly in inpatient care. They were described as *the next big thing* (provider, inpatient somatic care) and considered valuable for discussions with patients. Some indicators were considered relevant for inpatient care, such as mortality rates (outcome quality), minimum case numbers (structural quality), and staffing levels (structural quality). For outpatient care, adherence to clinical guidelines was emphasised. Experts also highlighted the importance of data on indication quality and the appropriateness of medical interventions, particularly in addressing the overprovision of care. A more detailed composition can be found in supplemental material 5 (Figure A 2).

### 3.2 Purpose of public reporting

In the public focus groups, participants expressed a strong desire for greater transparency and involvement through shared decision-making (see supplemental material 5, Figure A 3). One participant emphasised this frustration, stating: *Care and patient-centred, right? That's what everyone claims, but it doesn't happen at all.* (Patient, public). Another participant highlighted the belief that health data should be freely accessible to patients: *So, anything that has to do with us, we should have free access. The data belongs to me.* (Patient, public). Overall, the public saw transparency and shared decision-making as the key goals of public reporting, while provider choice was also named, but less often.

Experts had a more cautious view, seeing public reporting to reassure patients rather than provide detailed information: *As a canton, I simply want them [patients] to feel comfortable, feel safe and not worry about whether, if someone is on this [hospital planning] list. We are responsible for ensuring that they are good service providers. The population doesn't need to know much more than that.* (Regulator, inpatient somatic care). Similarly, in the outpatient focus group, some experts expressed scepticism about the importance of public reporting for patients: *And the patient doesn't really matter or is of secondary importance. They are the centre; they are in the way.* (Provider, outpatient somatic care). Some doubted whether patients could effectively interpret and use quality data, questioning its impact on decision-making.

### 3.3 Purpose of quality monitoring

Quality monitoring was seen as primarily aimed at continuous improvement in care (see supplemental material 5, Figure A 4). One inpatient somatic care provider emphasised: *And quality monitoring should primarily give us the opportunity to drive quality forward and check whether we're doing it successfully.* (Provider, inpatient somatic care). This sentiment is echoed by payers, who highlighted that the main objective of quality monitoring is to use the information gathered to drive improvements: *The main goal is to use quality information to make improvements. I said that's the main goal, I would say it's the only goal.* (Payer, inpatient somatic care). In the inpatient group, participants discussed the role of regulators in overseeing this process. Specifically, participants noted that regulators should be able to intervene if quality issues arise. Outpatient experts also emphasised quality monitoring's role in better clinical decision-making and adherence to guidelines.

### 3.4 Facilitators to effective implementation of public reporting and quality monitoring

All groups shared a few comments on facilitators for effective implementation of public reporting and quality monitoring. However, the public focus groups expressed a desire for a centralised place or person to help them navigate the health system. This includes understanding the insurance system, interpreting bills, finding the right provider, and receiving post-discharge support. The inpatient somatic care experts emphasised the need for greater collaboration within the system to reduce its fragmentation. They recognised routine data as a facilitator for both public reporting and quality monitoring, as it enables reliable information while keeping provider effort manageable. The outpatient somatic care focus group mentioned routine data and structured data collection as levers. They also discussed the potential of artificial intelligence to reduce the effort of collecting and evaluating data. A more detailed composition can be found in supplemental material 5 (Figure A 5).

### 3.5 Barriers to effective implementation of public reporting and quality monitoring

Participants identified several key barriers to the successful implementation of public reporting and quality monitoring, which were categorised into conceptual, systemic, socio-cultural, and technical/data-related challenges (see supplemental material 5, Figure A 6).

Conceptual barriers were primarily linked to concerns over the additional burden placed on providers. Experts expressed apprehension that public reporting and quality monitoring could divert attention from direct patient care, with providers feeling overwhelmed by the need to document additional information. Some stakeholders viewed quality monitoring and reporting as an obligation, which created resistance and reduced engagement.

Stakeholders attributed systemic barriers to the fragmented nature of the Swiss health system. The lack of coordination between cantonal and federal health initiatives meant providers faced differing data requirements from various institutions, creating inefficiencies and additional burdens. This fragmentation also contributed to the issue of small case numbers, particularly in rural areas, where hospitals had insufficient data to make meaningful comparisons. For the public, a significant systemic barrier was the perceived restricted freedom of choice due to the various insurance models.

Socio-cultural barriers were tied to the trust that patients and the public place in health professionals. Many public participants noted that their trust in physicians diminished the need for transparency and oversight. The belief that physicians “know best” often led to reluctance in accepting more transparency or questioning medical decisions. This sense of trust made patients less likely to engage with quality information or public reporting, as they felt it unnecessary. Similarly, in the inpatient somatic care group, experts from health associations emphasised that without fostering trust and open dialogue among providers, quality indicators alone would not drive meaningful improvements. This was further compounded by a general fear of transparency within the health system, particularly when it involved publicly disclosing data on individual providers.

Technical and data-related barriers included challenges with data collection, management, and dissemination. There was widespread frustration with the time lag in data dissemination, with many experts noting that current data, often years old, could render quality monitoring ineffective in guiding timely decisions. The lack of interoperability between data systems meant that data was often collected in inconsistent formats, such as free-text entries or PDFs, making it difficult to analyse or share efficiently. This technical fragmentation, combined with privacy concerns from the public, slows down the process of gathering and sharing actionable quality information.

### 3.6 Selection of quality indicators

Participants across all focus groups highlighted several criteria when it comes to selecting quality indicators (see Fig. 3). The public highlighted the importance of comprehensibility when it comes to quality indicators. For them, it is crucial that the indicators are easy to understand and transparent. In the inpatient somatic care focus groups, experts emphasised that quality indicators should be actionable, meaning they must help providers make improvements: *They are always looking for indicators that help me to change something* (Provider, inpatient somatic care). Additionally, experts stressed that indicators should prioritise data from key problem areas: *Therefore, the indicators that bring the greatest benefit for development should be selected. Where are the biggest problem areas and whether that is then process or outcome indicators or structure is the second question.* (Association representative, inpatient somatic care). Moreover, experts agreed that indicators need to be scientifically validated, ideally by an international audience, to ensure credibility and relevance.

**Fig. 3:**
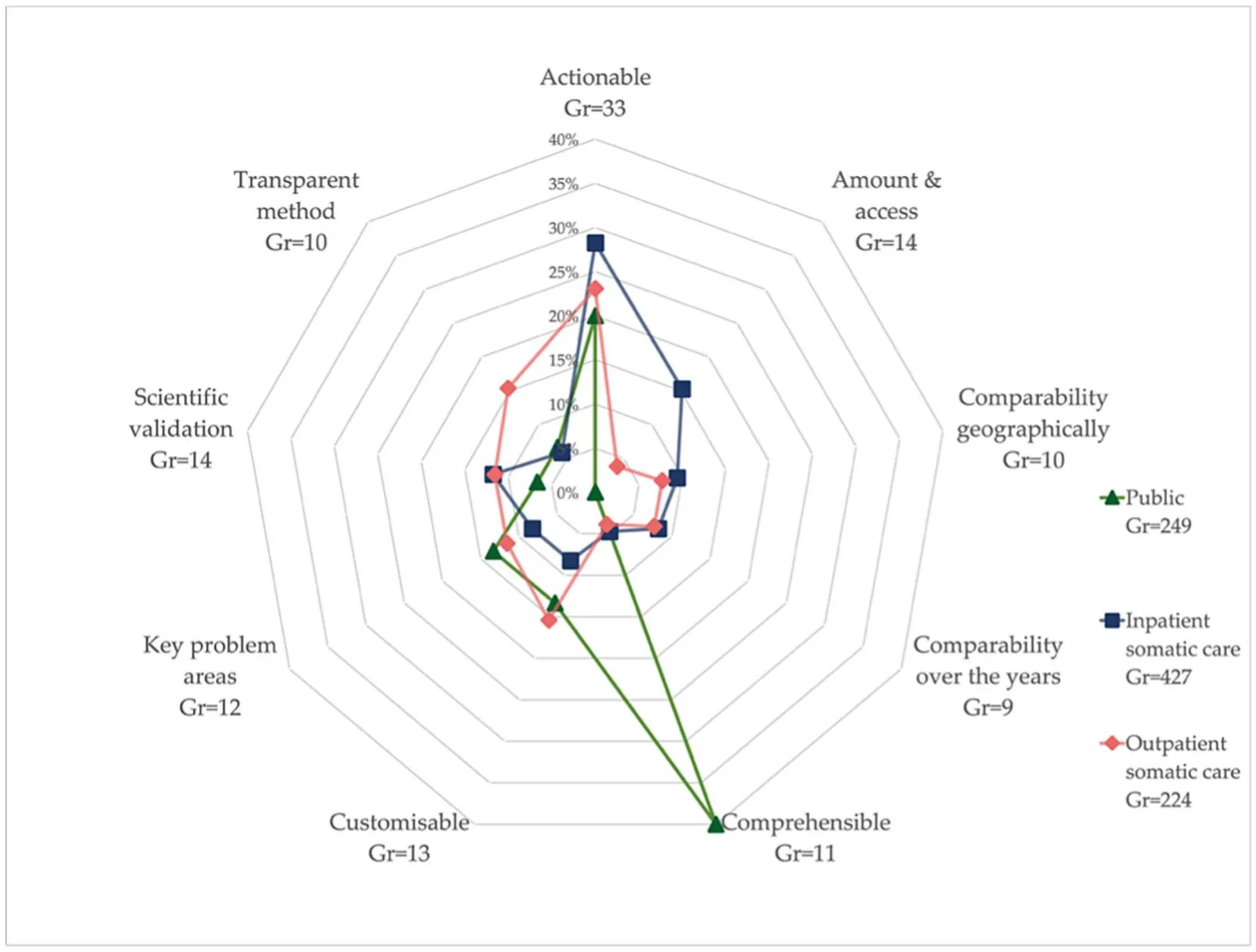
Overview of codes related to the selection of indicators for public reporting and quality monitoring displayed as a percentage of all the selection criteria discussed by focus groups. **Note:** Percentages represent the frequency of this theme relative to all topics discussed. Gr (Groundedness): Number of citations encoded by a code.

In the outpatient somatic care focus groups, experts shared similar views on the importance of actionable indicators that align with providers' practical needs. They emphasised the importance of customising indicators to stakeholder needs and making the data processing method transparent.

### 3.7 Synthesis

This study explored the quality information needs of stakeholders in Switzerland's inpatient and outpatient somatic care settings. The public emphasised the need for clear, accessible quality information, particularly for shared decision-making. Particularly, patients with chronic or severe illnesses emerged as highly knowledgeable about their conditions, often actively seeking to understand their symptoms, diagnoses, and treatment options. This may reflect a shift away from blind trust in physicians based solely on their professional status. An unmet need identified was the lack of a single point of contact to help patients navigate the health system and make appropriate choices. This gap is also reflected in their demand for quality information, with shared decision-making emerging as a key goal of public reporting and an area in which they desire greater access to relevant data.

Inpatient experts focused on quality monitoring to drive improvement, emphasising indicators like PROMs, PREMs, and mortality rates. They stressed the importance of using routine data and increasing collaboration within the health system, emphasising that fragmented data systems and a lack of coordination between providers hinder the effectiveness of quality monitoring and public reporting. Outpatient experts, while often sharing similar views with inpatient experts, also highlighted better clinical decision-making as a goal of quality monitoring. They were apprehensive about increased transparency, particularly when it involved publicly reporting provider-level data. Both groups agreed on the importance of actionable quality indicators.

## 4 Discussion

The findings confirm that stakeholder groups have distinct information needs.

### 4.1 Motivating public engagement in public reporting

The public seeks to understand their symptoms and diagnosis, own their health data, and make informed decisions. They primarily seek quality indicators that are clear, understandable, and relevant to their health decisions. However, experts do not regard the public as the primary audience for quality reporting, despite growing calls for transparency and shared decision-making. Our findings suggest that this debate may rest on an insufficient understanding of why patients seek quality information. Public participants named provider choice less often than transparency and shared decision-making as goals for public reporting. For patients, quality information may therefore serve less as a tool for selecting providers and more as a basis for informed discussions with their physicians. If public reporting is assessed solely by its effect on provider choice, its potential value for patients may be underestimated [33]. The misalignment, where patients are not seen as central figures in the health system, is a broader systemic issue observed globally, ranging from Australia [25] to Germany [18] to the US [34]. In Switzerland, existing public reporting initiatives such as Spitalfinder, Welches-Spital.ch, or Spitalinfo vary significantly in the type and quality of information presented, reflecting inconsistencies in transparency efforts [6]. Nonetheless, previous studies confirm that the public wishes to take a more active role in decision-making [35], and PROMs could enhance shared decision-making by facilitating communication between patients and providers [36]. Moreover, in Switzerland, a substantial share of the population reports difficulty in finding, understanding and applying health information [37]. Public reporting should therefore consider not only which information is published, but also how it reaches patients with lower health literacy, for example through a single point of contact as requested by our participants.

### 4.2 Enhancing transparency in quality monitoring

The inpatient somatic care discussions highlighted the relevance of PROMs, PREMs, indication quality, mortality rates, and staffing levels. In contrast, outpatient care providers prioritised indicators aligned with clinical guidelines but raised concerns about greater transparency, particularly around provider-level data. This reluctance may stem from the hierarchical and bureaucratic nature of health systems, which can foster a blame culture [38]. Shifting toward an open learning and reflection-oriented culture could help mitigate these concerns and encourage greater acceptance of provider-level monitoring. Additionally, Switzerland's inpatient somatic care sector has more experience with quality measurement and monitoring than the outpatient care sector [6]. These differing levels of maturity should be carefully considered when designing a national quality strategy.

### 4.3 Actionable quality indicators for continuous improvement

Providers from in- and outpatient somatic care focus on scientifically validated and actionable indicators that help improve quality. However, identifying specific quality issues from the available indicators requires considerable effort, leaving providers with less time to implement solutions. Experts in our study criticised that quality data are often several years old when they reach providers, which limits their use for timely decisions. This is consistent with evidence from audit and feedback research, which shows that feedback is most effective when it is timely, repeated and accompanied by improvement plans [39]. The improvement potential of quality monitoring can be further exploited if indicators are more actionable and help providers complete the quality improvement cycle [40].

### 4.4 A coordinated national strategy to overcome barriers in quality monitoring

The study also identified several facilitators and barriers to effective quality monitoring and public reporting. Facilitators include routine data use, increased collaboration among providers within the health system, and a growing emphasis on patient-centred care. However, significant barriers remain, including systemic fragmentation, technical challenges in standardising data collection, and privacy concerns. The administrative burden of data collection and documentation was emphasised by inpatient stakeholders, who feared increased workload without clear benefits. Multiple uncoordinated initiatives increase the burden on providers, as they must report data to various entities. These findings align with recent studies, which concluded that providers spend an unreasonable amount of time on quality data documentation, which is diverting time from patient care and from improving quality 11., 41.. Additionally, if providers do not perceive quality indicators as value-adding to care quality, they become less precise or omit to record quality indicators, leading to unreliable quality information [11]. First steps toward a more coordinated approach are underway in Switzerland: the Federal Quality Commission (FQC) is designing a national quality monitoring and public reporting system [27], and instruments such as QUALICATOR support a systematic collection and evaluation of existing quality indicators, which could help reduce redundant measurement and focus data collection on indicators that add value [14].

### 4.5 Strengths and limitations

The strengths of this study include the diverse perspectives of stakeholders. Focus groups were conducted across multiple Swiss regions to ensure geographic and cultural representation, and data analysis was carried out in the original languages, minimizing biases. The purposive sampling approach enabled the inclusion of relevant expert participants and diverse public perspectives. Furthermore, separating focus groups for the public and experts ensured that all participants could express their views freely. However, the sample size and voluntary participation mean the results are indicative and may not be generalizable. The discussions could have been biased by the content of the questions and the limited time allocated to topics. Social desirability bias, which is common in focus groups, may have influenced participants' responses. We addressed this by using neutral moderators (researchers) and carefully worded questions to encourage open and honest discussions. Additionally, confidentiality was ensured to reduce any pressure on participants. Self-selection bias may have occurred, as individuals with stronger opinions about quality information were more likely to participate. Furthermore, the inclusion of various expert stakeholders as a group prevents specific conclusions about individual stakeholder perspectives. The findings are rooted in the Swiss health system and may have limited applicability to other health systems.

### 4.6 Future research outlook

Future research should focus on developing and testing prototypes of quality monitoring and public reporting platforms that incorporate various indicators and formats. Further research, possibly using experimental designs, is needed to explore the role of patient engagement in public reporting, particularly regarding shared decision-making and the accessibility of information for non-experts.

## 5 Conclusion

This study highlights the complexity of designing quality monitoring and public reporting systems that address the diverse needs of stakeholders. While providers prioritise quality monitoring for internal improvements, the public increasingly demands transparency and greater involvement in decision-making. Defining the public as the primary audience for public reporting and promoting shared decision-making are essential to increasing the effectiveness and utilisation of these initiatives. A more coordinated national approach, integrating structured routine data collection and addressing concerns about transparency and administrative burden, could enhance the effectiveness of both public reporting and quality monitoring. Experts and patients should be closely involved in designing public reporting and quality monitoring systems to ensure that the right indicators are registered for improving health care quality. Last, public reporting and quality monitoring should be separated, giving stakeholders the relevant information.

## Declaration of generative AI in scientific writing

During the review of this work the authors used ChatGPT in order to shorten certain paragraphs and improve the readability and language of specific paragraphs in the manuscript. During the review phase, we used Consensus.ai to search for additional sources. After using this tool, the authors reviewed and edited the content as needed and take full responsibility for the.

## CRediT authorship contribution statement

**Carla Walker:** Writing – review & editing, Writing – original draft, Visualization, Software, Project administration, Methodology, Investigation, Formal analysis, Data curation, Conceptualization. **Maxime Sapin:** Writing – review & editing, Writing – original draft, Software, Methodology, Formal analysis, Data curation, Conceptualization. **Sarah Mantwill:** Writing – review & editing, Validation, Resources, Project administration, Methodology, Data curation, Conceptualization. **Justus Vogel:** Writing – review & editing, Validation, Resources, Project administration, Methodology, Investigation, Data curation, Conceptualization. **David Ehlig:** Writing – review & editing, Validation, Methodology, Data curation, Conceptualization. **Alexander Geissler:** Writing – review & editing, Validation, Supervision, Methodology, Conceptualization.

## Declaration of competing interest

The authors declare that they have no known competing financial interests or personal relationships that could have appeared to influence the work reported in this paper.
