# Supplementary material for: Empowering patients to choose and providers to improve. A focus group study on quality information needs in Switzerland's in- and outpatient somatic care
Source: Health Policy Open. 2026 Oct 16;11:100191. doi: 10.1016/j.hpopen.2026.100191 (PMC13717042; doi:10.1016/j.hpopen.2026.100191)
Supplement: Supplementary file 1 [file mmc1.docx]

**Supplemental material 1: Consolidated Criteria for Reporting Qualitative Research (COREQ)**

| **No. Item** | **Guide questions/description** | **Reported on Page #** |
| --- | --- | --- |
| **Domain 1: Research team and reﬂexivity** |  |  |
| *Personal Characteristics* |  |  |
| 1. Interviewer/facilitator | Which author/s conducted the interview or focus group? | See supplemental material 3 |
| 2. Credentials | What were the researcher’s credentials? E.g. PhD, MD | See supplemental material 3 |
| 3. Occupation | What was their occupation at the time of the study? | See supplemental material 3 |
| 4. Gender | Was the researcher male or female? | See supplemental material 3 |
| 5. Experience and training | What experience or training did the researcher have? | See supplemental material 3 |
| *Relationship with participants* |  |  |
| 6. Relationship established | Was a relationship established prior to study commencement? | See supplemental material 3 |
| 7. Participant knowledge of the interviewer | What did the participants know about the researcher? e.g. personal goals, reasons for doing the research | See supplemental material 3 |
| 8. Interviewer characteristics | What characteristics were reported about the interviewer/facilitator? e.g. Bias, assumptions, reasons and interests in the research topic | See supplemental material 3 |
| **Domain 2: study design** |  |  |
| *Theoretical framework* |  |  |
| 9. Methodological orientation and Theory | What methodological orientation was stated to underpin the study? e.g. grounded theory, discourse analysis, ethnography, phenomenology, content analysis | Page 3 |
| *Participant selection* |  |  |
| 10. Sampling | How were participants selected? e.g. purposive, convenience, consecutive, snowball | Page 2 |
| 11. Method of approach | How were participants approached? e.g. face-to-face, telephone, mail, email | Page 2 |
| 12. Sample size | How many participants were in the study? | Page 2 |
| 13. Non-participation | How many people refused to participate or dropped out? Reasons? | Page 2 |
| *Setting* |  |  |
| 14. Setting of data collection | Where was the data collected? e.g. home, clinic, workplace | Page 2  . |
| 15. Presence of non-participants | Was anyone else present besides the participants and researchers? | No |
| 16. Description of sample | What are the important characteristics of the sample? e.g. demographic data, date | Supplemental material 2 |
| *Data collection* |  |  |
| 17. Interview guide | Were questions, prompts, guides provided by the authors? Was it pilot tested? | Supplemental material 4 |
| 18. Repeat interviews | Were repeat interviews carried out? If yes, how many? | No repeat focus groups were carried out. Each participant took part in one focus group only. |
| 19. Audio/visual recording | Did the research use audio or visual recording to collect the data? | Page 3 |
| 20. Field notes | Were ﬁeld notes made during and/or after the interview or focus group? | Page 2-3 |
| 21. Duration | What was the duration of the interviews or focus group? | Page 2 |
| 22. Data saturation | Was data saturation discussed? | Page 3 |
| 23. Transcripts returned | Were transcripts returned to participants for comment and/or correction? | Page 3 |
| **Domain 3: analysis and ﬁndings** |  |  |
| *Data analysis* |  |  |
| 24. Number of data coders | How many data coders coded the data? | Page 3 |
| 25. Description of the coding tree | Did authors provide a description of the coding tree? | Page 3 – 4 |
| 26. Derivation of themes | Were themes identiﬁed in advance or derived from the data? | Page 3 |
| 27. Software | What software, if applicable, was used to manage the data? | Page 3 |
| 28. Participant checking | Did participants provide feedback on the ﬁndings? | Page 3 |
| *Reporting* |  |  |
| 29. Quotations presented | Were participant quotations presented to illustrate the themes/ﬁndings? Was each quotation identiﬁed? e.g. participant number | Pages 3-7 |
| 30. Data and ﬁndings consistent | Was there consistency between the data presented and the ﬁndings? | Pages 3-7 |
| 31. Clarity of major themes | Were major themes clearly presented in the ﬁndings? | Pages 3-7, supplemental file 5 |
| 32. Clarity of minor themes | Is there a description of diverse cases or discussion of minor themes? | Pages 3-7, supplemental file 5 |

Supplemental material 2: Participant characteristics of expert focus groups

In the following tables, further characteristics of the expert focus group participants are presented. The size of the focus groups was selected to ensure a balance between diverse opinions and a manageable group dynamic. Specifically, each group consisted of 5-6 participants, which allowed for meaningful interaction while preventing any one individual from dominating the conversation.

The provider group comprised both public and private providers of in- and outpatient somatic care. The regulators were cantonal health directorates or federal health agencies. The payer group included representatives of health insurances. In Switzerland, cantonal health directorates serve dual roles as both regulators and payers within the health system. In this paper, we classify cantonal health directorates primarily as regulators due to their significant authority in overseeing healthcare services, ensuring compliance with health regulations, and their role in health system governance. The Swiss healthcare associations included, for instance, associations of medical professionals or the association of Swiss health insurers.

Table A 1: Overview of experts by healthcare area and organization category

| **Healthcare area** | **Organization (descriptive)** | **Category** |
| --- | --- | --- |
| Inpatient somatic care | Quality Expert, University Hospital | Provider |
|  | Associate Physician & Lecturer, University Health Institute | Provider |
|  | Quality Manager, Regional Hospital | Provider |
|  | Head of Quality, Clinical Safety & Process Management, University Hospital | Provider |
|  | President, National Nursing Leadership Association / Nursing Director, Hospital | Provider |
|  | Head of Hospital Administration, Public Health Service, Cantonal Health Directorate | Regulator |
|  | Legal Advisor, Cantonal Health Directorate | Regulator |
|  | Quality Manager, Cantonal Health Directorate | Regulator |
|  | Data Management and Statistics Specialist, Federal Health Agency | Regulator |
|  | Specialist in Medical Standards, Health Insurance Company | Payer |
|  | Head of Acute Care, Deputy Management, National Quality Development Association | Association |
|  | Project Manager, Quality & Health Technology Assessment, Health Insurers Association | Association |
|  | Head of Policy & Deputy Director, National Health Insurance Association | Association |
|  | Project Manager, Quality & Patient Safety, National Hospital Association | Association |
|  | Head of Advisory Services & Specialist in Internal Medicine, Patient Advocacy Organization | Association |
|  | Professor, Institute for Social and Preventive Medicine, University | Academic |
| Outpatient somatic care | Physiotherapist & Representative, Medical Quality Committee, Healthcare Provider Network | Provider |
|  | Co-Chief Physician, Orthopedics & Trauma Surgery, Head of Sports Medicine, Regional Hospital | Provider |
|  | Medical Network Manager & Executive Board Member, Physician Network | Provider |
|  | CEO, Health Research & Consulting Firm | Provider |
|  | Head of Integrated Care, Health Insurance Organization | Payer |
|  | Managing Director, Independent Quality Certification Foundation | Association |
|  | Vice President, National Association of Medical Practice Professionals | Association |
|  | Expert in Medical Service Quality, Health Insurance Association | Association |
|  | Expert in Healthcare Tariffs, National Pharmacy Association | Association |
|  | Head of Primary Care Research & Senior Physician, University Institute of Family Medicine / Representative, General Internal Medicine Society | Academic |

**Supplemental material 3: Research team & reflexivity**

All six authors (two female, four male) were involved in conducting and/or observing the focus groups. CW (female), a first-year PhD student and research assistant, acted as notetaker in the public and inpatient focus groups. MS (male), also a first-year PhD student and research assistant, participated in all focus groups as either notetaker or moderator. SM (female), a postdoctoral researcher with advanced training in qualitative research, served as a moderator in the public and expert focus groups. DE (male), a postdoctoral researcher and scientific project leader, moderated one of the outpatient somatic care focus groups. JV (male), also a postdoctoral researcher and scientific project leader, and AG (male), a professor and senior researcher, moderated across the full set of focus groups.

All researchers had at least basic academic training in qualitative methods, with SM having additional in-depth experience and formal training in qualitative research.

No prior personal relationships existed between researchers and participants. Before the focus groups, participants were informed about the study objectives and the project team during an online information session. In addition, expert participants were invited to an informal dinner on the evening prior to their respective focus group to foster open dialogue and engagement. Each focus group began with a 30-minute introductory session, in which the researchers presented the background of the project and provided participants with an overview of key concepts related to public reporting and quality monitoring in the Swiss healthcare system. Researchers introduced themselves and their roles at the beginning of each session. General information about the research team’s background and interest in the topic was shared, but personal motivations were not discussed.

Supplemental material 4: Questions for in- and outpatient focus groups

The following questions were used for moderators of the expert focus groups to guide the discussion.

1. From your perspective, what quality information needs to be reported for psychiatric care in Switzerland? (IF USEFUL SPECIFY: Considering your work environment or daily work, which quality information would be relevant to you?)

2. What are the most important criteria for the selection of quality indicators in your view?

PROMPTS FOR FACILITATORS, ordered according to importance:

- In your area / From your expertise, which quality information that would be needed is currently not available? How can this information be made available?
- Considering the Swiss healthcare system, what needs to be considered (in contrast to other countries)? Follow-up: What are current issues/obstacles in the Swiss system?
- Who should have access to quality information?
- From a methodological point of view, what are, in your view, important aspects to consider? Follow-up: Is risk adjustment important? According to which factors should be adjusted?
- How to deal with small sample sizes?
- At which aggregation level should information be made available?
- How should information be visualized? Follow-up: Should a data download be available?
- Who should conduct quality monitoring and to what purpose? Are there limits to quality monitoring?
- Who will use public reporting and for what purpose? Are there obstacles for using public reporting?

Supplemental material 5: Further subcode groups

Quality indicators for the public

The spider diagram below provides an overview of the subcodes under the category "quality indicators" and the relative mentions across the public focus groups.

Figure A 1: Overview of codes related to information needs of the public displayed as a percentage of all quality indicators discussed

Quality indicators for the experts

The spider diagram below provides an overview of the subcodes under the category "quality indicators" and the relative mentions across the in- and outpatient somatic care focus groups.

Figure A 2: Overview of codes related to information needs of the experts displayed as a percentage of all quality indicators discussed by the in- and outpatient somatic care focus groups

Purpose of public reporting

The spider diagram below provides an overview of the subcodes under the category "Purpose of public reporting" and the relative mentions across all three focus groups.

Figure A 3: Overview of codes related to the purpose of public reporting displayed as a percentage of all purposes discussed by focus groups

Purpose of quality monitoring

The spider diagram below provides an overview of the subcodes under the category "Purpose of quality monitoring" and the relative mentions across the in- and outpatient somatic care focus groups.

Figure A 4: Overview of codes related to the purpose of quality monitoring displayed as a percentage of all purposes discussed by in- and outpatient somatic care focus groups

Facilitators for public reporting and quality monitoring

The spider diagram below provides an overview of the subcodes under the category "Facilitators" and the relative mentions across all focus groups.

Figure A 5: Overview of codes related to facilitators of public reporting and quality monitoring displayed as a percentage of all facilitators discussed by focus groups

Overview of barriers

The spider diagram below provides an overview of the barrier categories and the relative mentions across all focus groups.

Figure A 6: Overview of codes related to barrier categories of public reporting and quality monitoring displayed as a percentage of all barriers discussed by focus groups
